# Two New Sulfate-Modified Dibenzopyrones With Anti-foodborne Bacteria Activity From Sponge-Derived Fungus *Alternaria* sp. SCSIOS02F49

**DOI:** 10.3389/fmicb.2022.879674

**Published:** 2022-05-04

**Authors:** Yaping Chen, Chuanna Liu, Kaliaperumal Kumaravel, Lihong Nan, Yongqi Tian

**Affiliations:** ^1^College of Pharmacy, Fujian University of Traditional Chinese Medicine, Fuzhou, China; ^2^College of Biological Science and Engineering, Fuzhou University, Fuzhou, China; ^3^Centre for Drug Discovery, Satyabhama University, Chennai, India

**Keywords:** sponge-derived fungi, *Alternaria* sp., sulfate-modified dibenzopyrones, anti-foodborne bacteria activity, antibacterial mechanism

## Abstract

At present, foodborne diseases (FBDs) caused by bacteria are gradually increasing every year, and the development of new antibiotics is an urgent necessity for human beings. To find novel antibacterial compounds, three sponge-derived fungal strains (SCSIOS02F40, F46, and F49) were investigated. As a result, *Alternaria* sp. SCSIOS02F49 was selected for investigation on its secondary metabolites because its ethyl acetate (EtOAc) extract of potato dextrose broth (PDB) culture showed rich metabolites and strong antibacterial activity. Two new dibenzopyrones with rare sulfate group (**1**–**2**), together with 10 known compounds (**3**–**12**), were isolated from the *Alternaria* sp. SCSIOS02F49. Their structures were confirmed by nuclear magnetic resonance (NMR), mass spectrometry (MS) data, and comparison with data from the relevant literature. Almost all compounds showed moderate inhibitory activity against eight foodborne bacteria (FBB) with minimum inhibitory concentration (MIC) values in the range of 15.6–250 μg/ml, and minimum bactericidal concentration (MBC) values in the range of 31.3–250 μg/ml. The antibacterial mechanism of compound 1 was preliminarily investigated using growth curves, scanning electron microscopy (SEM), and flow cytometry (FCM), which revealed that compound 1 altered the external structure of *Staphylococcus aureus* and caused the rupture or deformation of the cell membranes. This research provides lead compounds for the development of new antibiotics or microbial preservatives.

## Introduction

As a growing public health problem throughout the world, foodborne diseases (FBDs) have resulted in almost 1 in 10 people being ill, leading to 420,000 deaths every year (Chandankere et al., 2020; Li et al., 2020). Among them, FBDs caused by *Campylobacter pylori, Staphylococcus aureus, Listeria monocytogenes, Escherichia coli, Vibrio parahaemolyticus, Salmonella cholerae, Proteus mirabilis, Shigella flexneri*, and other foodborne bacteria (FBB) have been widely reported and are of great concern (Tack et al., 2019, 2020). The most common and effective measure for the prevention of FBB was chemical control. However, the frequent use of chemical preservatives or antibiotics had an increased risk of drug resistance (Medjeldi et al., 2018; Qais et al., 2019). Therefore, the development of new agents against bacteria without risk and harmful side effects is an urgent necessity for human beings.

In recent years, marine fungi have received widespread attention because of their tremendous capacity to produce structurally unique compounds with a broad range of pharmacological properties (Radic and Strukelj, 2012; Pang et al., 2016; Fang et al., 2021; Guo et al., 2021). Over the last 5 years, discoveries of new compounds from marine fungi have outpaced discoveries from all other marine phyla. In previous studies, the most explored marine fungi were *Penicillium* and *Aspergillus*. Recently, novel compounds isolated from marine fungi, such as *Acremonium, Alternaria, Pseudotrichosporon, Chaetomium*, and *Cladosporiosis*, have been reported. Their biological activities mainly involve antibacterial, anti-inflammatory, anti-virus, anti-tumor, anti-fouling, enzyme inhibition, etc (Carroll et al., 2019, 2020, 2021).

**Figure 1:**
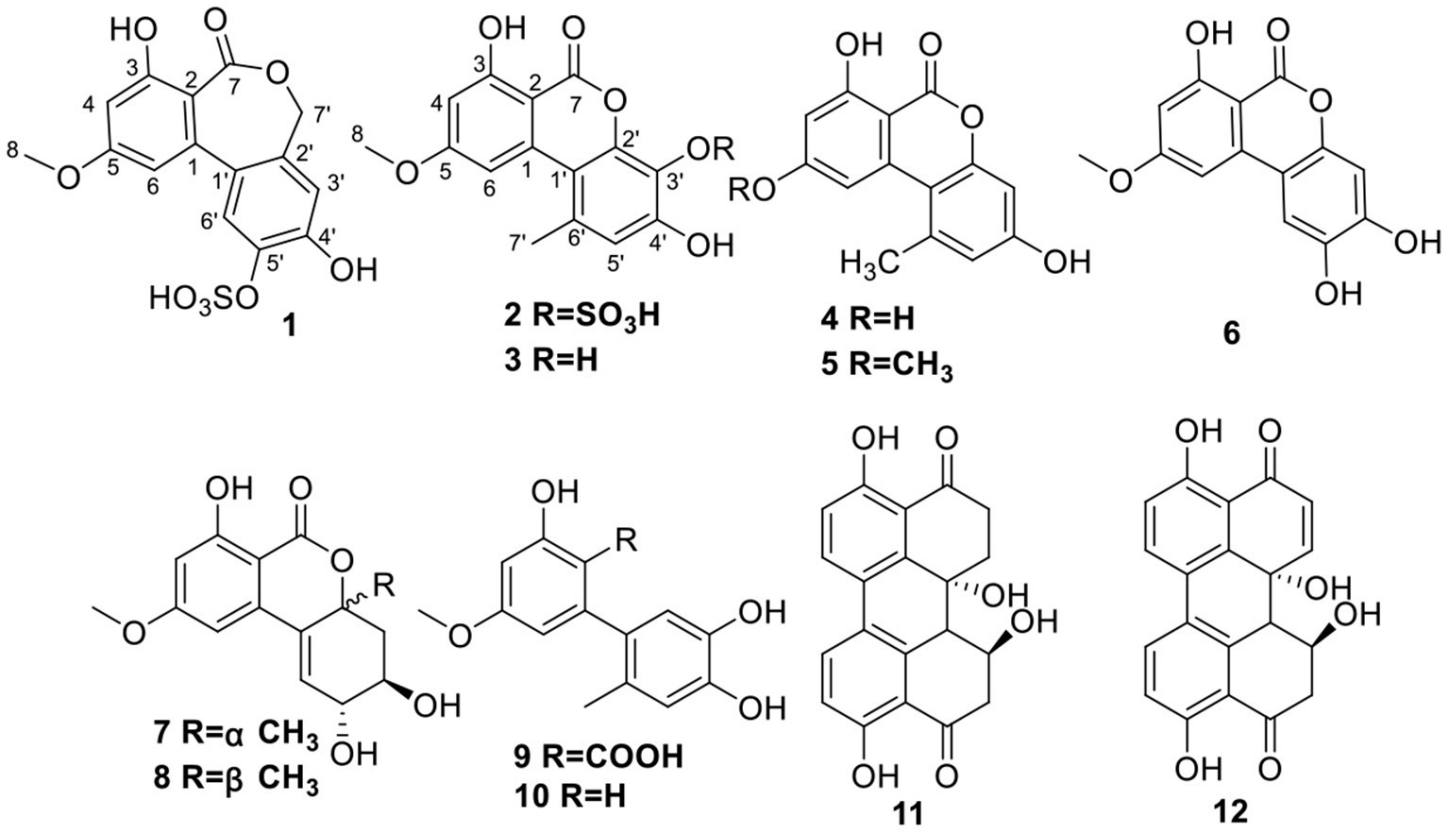
Structures of compounds **1**–**12**.

As a continuing effort in the search for new bioactive metabolites from the endophytic fungi (Tian et al., 2015a,b, 2018a,b,c; Cong et al., 2020), three fungal strains isolated from a marine sponge were subjected to chemical [Thin layer chromatography (TLC) and high pressure liquid chromatography (HPLC)] and antibacterial activity screening. *Alternaria* sp. SCSIOS02F49 was then selected as the target strain for large-scale fermentation and further research because of its abundant metabolites and strong antibacterial activity. Two rare sulfate-modified dibenzopyones, Alterlactone 5′-*O*-sulfate (**1**) and 3′-hydroxyalternariol 5-*O*-methyl ether-3′-*O*-sulfate (**2**), together with 10 known compounds were isolated from the *Alternaria* sp. SCSIOS02F49 (Figure 1). The antibacterial activities of all compounds against eight FBB were evaluated, and the antibacterial mechanism of **1** was preliminarily investigated. Details of the isolation, structure elucidation, and bioactivity screening of these metabolites are reported herein.

## Materials and Methods

### General Experimental Procedures

The nuclear magnetic resonance (NMR) spectra were recorded on a Bruker AC 500 (600) M NMR (Bruker, Fällanden, Switzerland) spectrometer with tetramethylsilane (TMS) as an internal standard. HRESIMS data were measured on an Agilent 6550iFunnelQ-TOFLC/MS (Bruker, Fällanden, Switzerland). UV spectra were recorded on a Shimadzu UV-2600 UV-Vis spectrophotometer (Shimadzu, Kyoto, Japan). Semi-preparative reversed-phase HPLC [(SP-RP) HPLC] was performed on a YMC-Pack Pro C_18_ *RS* column (5 μm, 250 × 10 mm id; YMC, Kyoto, Japan) with a High-Performance Liquid Chromatograph Primaide equipped with a photodiode array (PDA) detector. Silica gel GF254 used for TLC was supplied by the Qingdao Marine Chemical Factory, Qingdao, China. Sephadex LH-20 gel (GE Healthcare, Uppsala, Sweden) was used. Flow cytometer (FCM) (Accuri C6 Plus, Becton, USA) and scanning electron microscope (SEM) (JSM-6700F, JEOL, Japan) were used in the research of antibacterial mechanism. Spots were detected on TLC under UV light or by heating by spraying with 12% H_2_SO_4_ in H_2_O.

### Microbial Strains

Fungal strains were isolated from the sponge *Callyspongia* sp., which was collected from the sea area near Xuwen County (20.3265° N, 110.1750° E), Guangdong Province, China. The fungi SCSIOS02F40, F46, and F49 were identified as *Aspergillus* sp., *Didymellaceae* sp., and *Alternaria* sp., respectively, using a molecular biological protocol by DNA amplification and sequencing of the internally transcribed spacer (ITS) region (GenBank accession number KT164776, KU361223, and KU361224) (Supplementary Table 3). These three strains (MMPC NO. 5035-5037) were deposited at the Marine Microorganism Preservation Center, CAS Key Laboratory of Tropical Marine Bio-resources and Ecology, China. Bacteria strains of *Staphylococcus aureus* (ATCC25923), *Listeria monocytogenes* (CICC21633), *Escherichia coli* (ATCC25922), *Proteus mirabilis* (CMCC49005), *Vibrio parahaemolyticus* (ATCC17802), *Pseudomonas fluorescens* (ATCC13525), *Salmonella choleraesuis* (CICC13312), and *Shigella flexneri* (CMCC51571) were preserved in the Institute of Food and Marine Biological Resources, Fuzhou University.

### Fermentation, Extraction, and Isolation

Strain **F49** was cultured on Malt Broth-agar (MB-agar) plates at 25°C for 7 days. The seed medium (consisted of malt extract: 15 g, sea salt: 10 g, distilled water: 1,000 ml, pH: 7.4–7.8) was inoculated with strain **F49** and incubated at 25°C for 72 h on a rotating shaker (170 rpm). The seed solution (10 ml) was then incubated on a rotary shaker (170 rpm) at 25°C for 15 d in 1,000 ml × 30 conical flasks containing the liquid medium (300 ml/flask) composed of 40 g of potato, 4 g of glucose, 2.5 g of sea salt, and 200 ml pure water. The fermented whole broth (9 L) was filtered through cheesecloth to separate it into filtrate and mycelia. The filtrate was concentrated under vacuum to about a quarter of the original volume and then extracted three times with EtOAc to give an EtOAc solution, while the mycelia were extracted three times with acetone. The acetone solution was evaporated under reduced pressure to afford an aqueous solution. The aqueous solution was extracted three times with EtOAc to give a residual extract. Both the EtOAc solutions were combined and concentrated under vacuum to give a final EtOAc extract (16.43 g). The EtOAc extract was subjected to silica gel column chromatography (CC) eluted with petroleum ether/EtOAc in a gradient eluent (v/v, 50:1, 20:1, 10:1, 5:1, 2:1, 1:1, 0:1) to obtain 12 fractions (fractions 1–12) on the basis of TLC. Fraction 10 (fr. 10) was subjected to Octadecylsilyl (ODS) chromatography eluted with MeOH/H_2_O in a gradient eluent (1:9, 2:3, 3:2, 4;1, 9:1), to give 4 subfractions (fr. 10.1–10.4). Fr. 10.2 was further purified by (SP-RP) HPLC eluting with CH_3_CN-H_2_O (15:85) to afford **1** (22.7 mg). In the same way, compounds **2** (8.4 mg) and **3** (6.4 mg) were purified from fr. 10.3 by (SP-RP) HPLC [25% methyl cyanide (MeCN), 75% H_2_O + 1‰ trifluoroacetic acid (TFA)]. Fr. 6 was purified by Sephadex LH-20 (CH_3_Cl/MeOH, 1:1) to give 3 subfractions (fr. 6.1–6.3). Fr. 6.1 was further purified on (SP-RP) HPLC by 35% MeCN to give **4** (8.4 mg), **5** (10.1 mg), and **6** (10.8 mg). Compounds **7** (9.3 mg), **8** (28.9 mg), **9** (2.3 mg), and **10** (3.7 mg) were purified from Fr. 6.2 by (SP-RP) HPLC (35% MeCN). Fr. 6.3 was further purified on (SP-RP) HPLC by 25% MeCN to give **11** (3.7 mg) and **12** (4.5 mg).

Alterlactone 5′-*O*-sulfate (**1**): Brown needle crystal; UV (MeOH) λ_max_ (log ε) 203 (4.34), 253 (4.08), 286 (3.67) nm (Supplementary Figure 8); HRESIMS *m/z* 367.0126 [M - H]^−^ (calcd for C_15_H_12_O_9_S, 368.01985) (Supplementary Figure 7); ^1^H and ^13^C NMR data, Table 1.

**Table 1:** ^1^H NMR (500 MHz), ^13^C NMR (125 MHz), HMBC, and NOESY data of compounds **1** and **2** in DMSO-*d*_6_.

**No**.	**1**				**2**			
	**δ_H_**	**δc, type**	**HMBC**	**NOESY**	**δ_H_**	**δc, type**	**HMBC**	**NOESY**
1		140.1, C				138.2, C		
2		109.7, C				98.9, C		
3		160.8, C				164.6, C		
4	6.54, d (2.5)	101.4, CH	2, 3, 5, 6, 7	8	6.66, d (2.0)	100.1, CH	2, 3, 5, 6, 7	8
5		162.9, C				166.7, C		
6	6.46, d (2.5)	105.8, CH	2, 4, 5, 7, 1′	8, 6′	7.27, d (2.0)	104.1, CH	2, 4, 5, 7, 1′	8, 7′
7		169.1, C				164.8, C		
8	3.83, s	55.9, CH_3_	5		3.93, s	56.3, CH_3_	5	
1′		130.1, C				110.5, C		
2′		132.1, C				145.9, C		
3′	7.03, s	117.3, CH	1′, 4′, 5′, 7′	7′		127.5, C		
4′		150.2, C				151.4, C		
5′		142.5, C			6.86, s	118.8, CH	1′, 3′, 4′, 7′	
6′	7.56, s	123.0, CH	1, 1′, 2′, 4′, 5′	6		133.7, C		
7′	4.92, d (10.9)	68.1, CH_2_	7, 1′, 2′, 3′	3′	2.73, s	25.2, CH_3_	1, 6, 1′, 3′, 5′, 6′	
	4.90, d (10.9)							
4′-OH	10.30, brs^a^				9.70, s		3′, 4′, 5′	
5′-OH	9.40, brs^a^							
3-OH	4.14, brs^a^				11.82, s		2, 3, 4, 5	

a *Assignments may be interchanged*.

3'-hydroxyalternariol 5-*O*-methyl ether-3′-*O*-sulfate (**2**): Yellow amorphous solid; UV (MeOH) λ_max_ 203 (4.48), 217 (4.42), 258 (4.55), 287 (3.94), 298 (3.90), 340 (3.89) nm (Supplementary Figure 16), HRESIMS *m/z* 367.0120 [M - H]^−^ (calcd for C_21_H_25_O_6_, calcd for C_15_H_12_O_9_S, 368.01985) (Supplementary Figure 15); ^1^H and ^13^C NMR data, Table 1.

### Antibacterial Bioassay

The diameter of the inhibition zone (DIZ) of compounds was evaluated according to the previously published literature (Yao et al., 2011). In short, 50 μ*l* of compounds solutions at 500 μg/mL were injected into the small round hole (diameter of 6 mm) of LB agar medium containing bacterial suspension. After incubation at 37°C for 12 h, the DIZ was measured with a digital caliper. The minimum inhibitory concentration (MIC) of compounds was carried out with a 2-fold dilution method in a 96-well plate, and the minimum bactericidal concentration (MBC) of the compounds was determined with a suitably modified plate coating method (Wang et al., 2021). The brief process is as follows: The bacterial suspension (50 μ*l*) and 50 μ*l* of compounds solutions at different concentrations (the final concentration series is 0.12~250 μg/ml) were added to the 96-well plate. After 12 h of incubation at 37°C, the turbidity of the culture medium in each well was visually observed, and the final concentration of the dilution mass corresponding to the previous well, where the culture medium became turbid, was determined as the MIC value of compounds. According to the results of the MIC, 15 μ*l* of the culture solution was aspirated and spread over the solid growth medium, which was incubated at a constant temperature in an incubator at 37°C for 24 h. The final concentration of the diluted mass is regarded as the MBC value of the test compounds. The positive control was tetracycline, and the blank group was a solvent that dissolves the samples.

### Antibacterial Mechanism

#### Growth Curves Analysis

The growth curves of **1** against *S. aureus* were measured using the reported method (Lin et al., 2021). The bacterial suspension (1 × 10^6^ CFU/ml) and LB medium containing the tested compounds were mixed at ultimate concentrations of 0.5 × MIC, 1.0 × MIC, and 2.0 × MIC into each well on a 96-well plate. After cultivating at 37°C, bacterial growth was monitored by the absorbance at 600 nm with the microplate reader, and OD600 nm values were acquired at selected time points (0, 1, 2, 3, 4, 5, 6, 7, 8, 9, 10, 11, 12, and 24 h).

#### SEM Analysis

Scanning electron microscopy was performed according to the method of a previously published manuscript (Lin et al., 2021). The logarithmic *S. aureus* was washed two times with polybutylene succinate (PBS) (pH 7.4, 0.1 M), then resuspended to a concentration of about 1 × 10^7^ CFU/ml. The bacterial suspension was added into the medium containing 1.0 × MIC and 2.0 × MIC tested compounds and incubated at 37°C and 180 rpm for 6 h. The culture was centrifugated (3,000 rpm, 10 min), washed with 0.1 M PBS three times, and stabilized with 2.5% glutaraldehyde overnight at 4°C in the dark. Samples were dehydrated with gradient concentration (30, 40, 50%) ethanol, covered on a silicon wafer (5 mm × 5 mm), and dried naturally at room temperature. After spraying gold, the samples were observed and analyzed by SEM.

#### FCM Analysis

Flow cytometry analysis was conducted following the methodology of the literature (Du et al., 2018) with slight modifications. The logarithmic *S. aureus* (1 × 10^7^ CFU/ml) was added into the medium containing 0.5 × MIC, 1.0 × MIC, 2.0 × MIC, and 4.0 × MIC tested compounds. All cultures (sample group and control group) were incubated at 37°C and 180 rpm for 2 h. The culture of 1 ml was washed with 0.1 M PBS (pH 7.4) and added with 2 μ*l* of propidium iodide (PI) at room temperature in the dark for more than 40 min. Finally, the FCM was used for detection and analysis.

## Results

### Selection of Target Strain

**Figure 2:**
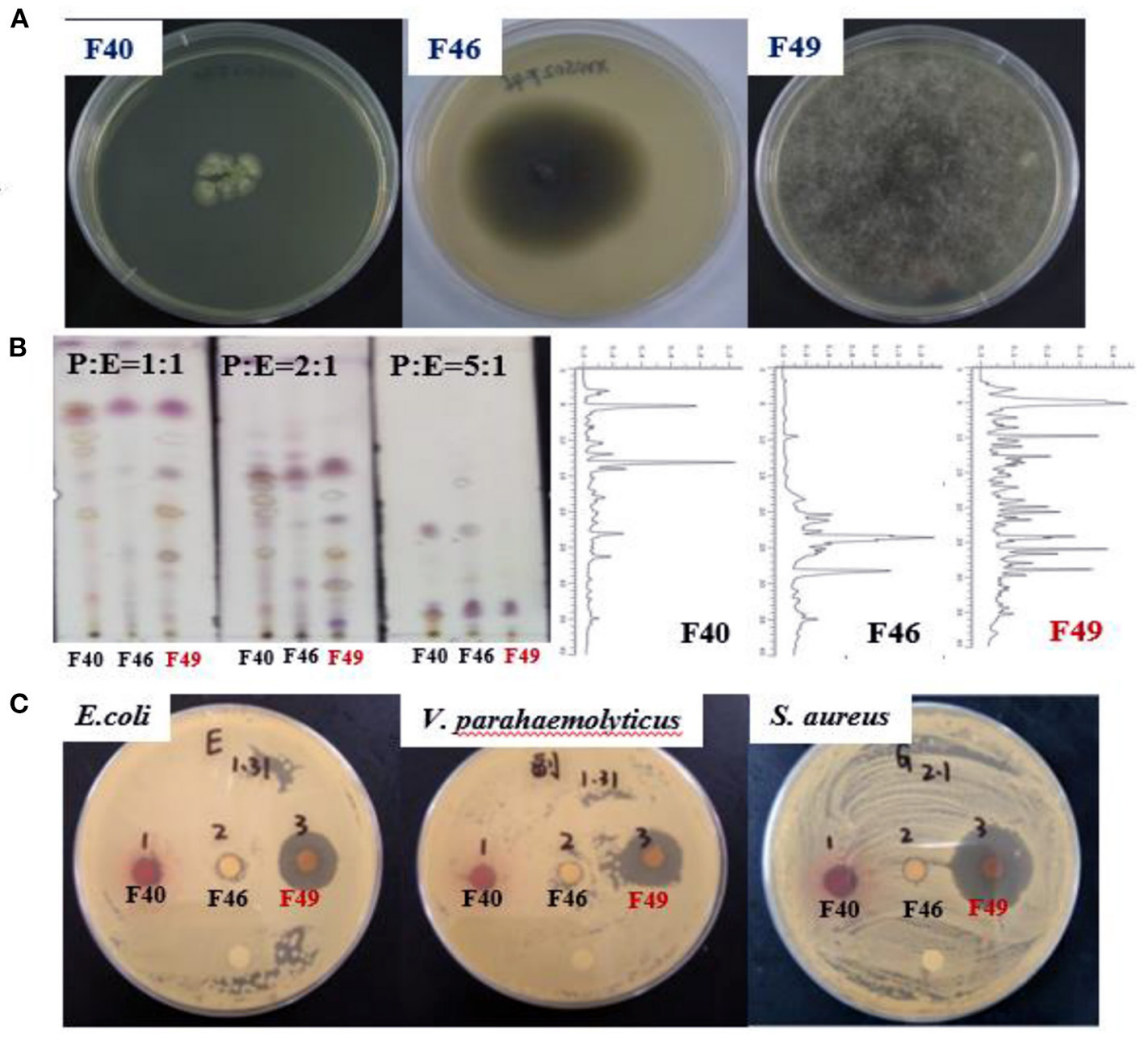
Selection of the target strain. **(A)** Three strains; **(B)** Chemical screening [Thin layer chromatography (TLC) and high pressure liquid chromatography (HPLC)]; **(C)** Antibacterial activity screening.

Chemical screening (TLC and HPLC) and antibacterial activity screening were used to screen the three marine fungal strains, **F40**, **F46**, and **F49** (Figure 2A). TLC analysis and HPLC fingerprint analysis (Figure 2B) showed that the EtOAc extract of potato dextrose broth (PDB) culture of **F49** contained abundant and moderately polar secondary metabolites. Meanwhile, the results of antibacterial activity screening (Figure 2C) showed that the EtOAc extract of PDB culture of **F49** (500 μg/ml) showed strong activity against three FBB (*E. coli, S. aureus*, and *V. parahaemolyticus*). Therefore, **F49** was selected as the target strain for large-scale fermentation and further research, because of its abundant metabolites and strong antibacterial activity.

### Structure Identification

**Figure 3:**
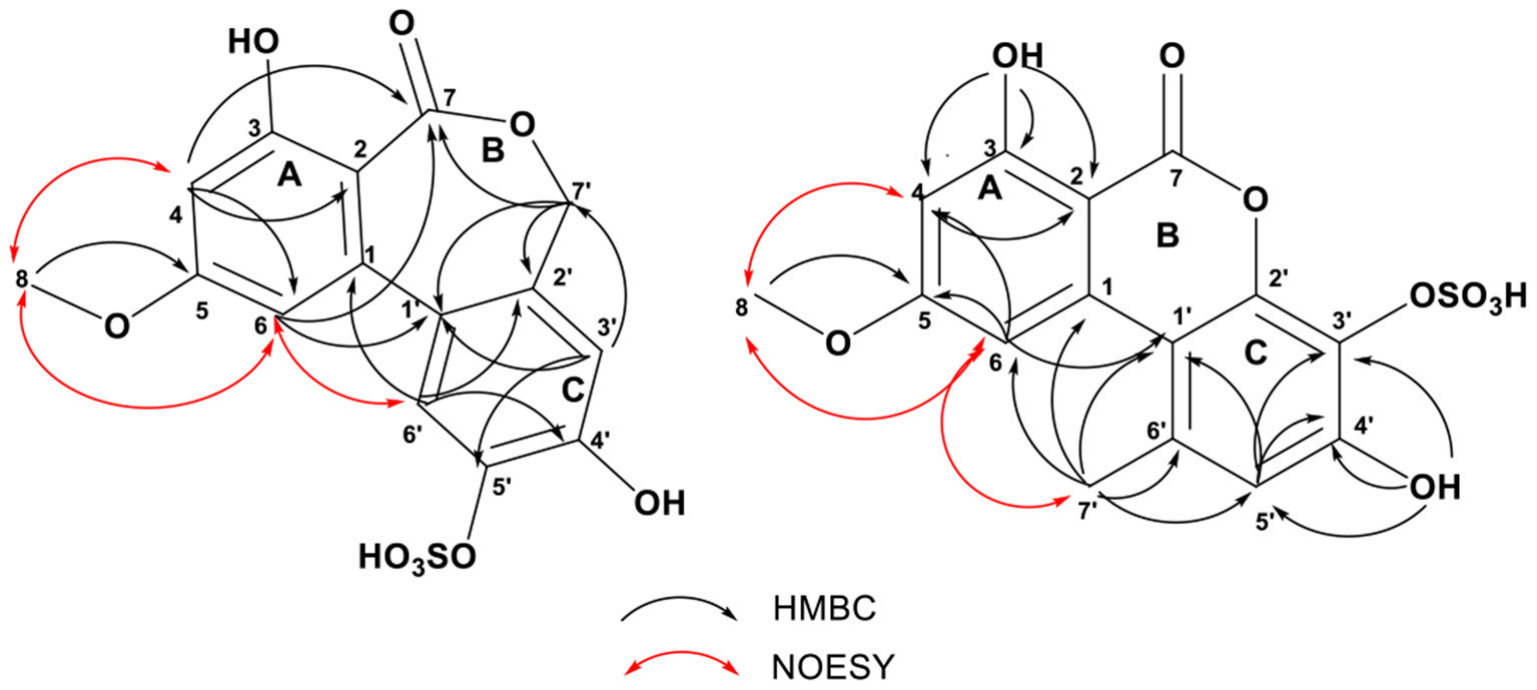
Selected HMBC and NOESY correlations of compounds **1** and **2**.

Compound **1** was obtained as a brown needle crystal. The molecular formula C_15_H_12_O_9_S was determined by its HRESIMS at *m/z* 367.0126 [M–H]^−^, indicating 10 degrees of unsaturation (Supplementary Figure 7). The ^1^H NMR spectrum showed presence of one methoxy [δ_H_ 3.83 (s, H_3_-8)], one oxygenated methylene [δ_H_ 4.92 (d, *J* = 10.9 Hz, H-7'a), 4.90 (d, *J* = 10.9 Hz, H-7′b)], four olefinic protons [δ_H_ 6.54 (d, *J* = 2.5 Hz, H-4), 6.46 (d, *J* = 2.5 Hz, H-6), 7.03 (s, H-3'), 7.56 (s, H-6′)], and three exchangeable protons (δ_H_ 10.11, 11.96, 4.14) (Supplementary Figure 1). Analysis of the ^13^C NMR, DEPT, and HSQC spectroscopic data of **1** revealed 15 carbon signals, involving one methoxy (δ_C_ 55.9, CH_3_-8) and one methylene (δ_C_ 68.1, CH_2_-7′), four olefinic methines (δ_C_ 101.1, CH-4; δ_C_ 105.8, CH-6; δ_C_ 117.3, CH-3′; δ_C_ 123.0, CH-6'), four oxygenated quaternary carbons (δ_C_ 160.8, C-3; δ_C_ 162.9, C-5; δ_C_ 150.5, C-4'; δ_C_ 142.5, C-5′), one carboxyl carbon (δ_C_ 169.1, C-7), and four olefinic quaternary carbons (δ_C_ 140.1, C-1; δ_C_ 109.7, C-2; δ_C_ 130.1, C-1', δ_C_ 132.1, C-2′) (Table 1 and Supplementary Figures 1–4). The HMBC correlations of H_2_-7' (4.92, d, *J* = 10.9 Hz; 4.90, d, *J* = 10.9 Hz)/C-7 (δ_C_ 169.1), C-1′ (δ_C_ 130.1), C-2' (δ_C_ 132.1), and C-3′ (δ_C_ 117.3) indicated that the lactone ring (B ring) in **1** was not a common six-membered ring, but instead was a rare seven-membered ring, which formed through a hydroxylated methyl group (Table 1, Figure 3, and Supplementary Figure 5). These signals were closely related to those of alterlactone (Aly et al., 2008), except that the chemical shift of C-5′ in **1** appeared at a lower field (δ_C_ 142.5) than in alterlactone (δ_C_ 146.6). Combining MS, HMBC, and NOESY spectrum, it was easy to speculate a sulfate substituent at C-5′ in **1** (Figure 3 and Supplementary Figures 5–7). Thus, **1** was elucidated and named alterlactone 5′-*O*-sulfate.

Compound **2** possessed the same molecular formula as **1** (m/z 367.0126 [M–H]^−^, C_15_H_12_O_9_S). Comprehensive analysis of the ^1^H, ^13^C NMR, DEPT, and HSQC spectra revealed the presence of one methyl [δ_H_/δ_C_ 2.73 (s)/25.2], one methoxy [δ_H_/δ_C_ 3.92 (s)/56.3], three olefinic methines [δ_H_/δ_C_ 6.66 (d, *J* = 2.0 Hz)/100.1; 7.27 (d, *J* = 2.0 Hz)/104.1; 6.85 (s)/118.8], ten quaternary carbons (δ_C_ 98.9, 110.5, 127.5, 133.7, 138.2, 145.9, 151.4, 164.6, 164.8, 166.7), and two exchangeable protons (δ_H_ 11.83, 9.70) (Table 1 and Supplementary Figures 9–12). These signals were closely related to those of 3′-hydroxyalternariol 5-*O*-methyl ether (**3**) (Supplementary Table 1 and Supplementary Figures 17, 18), except for the chemical shift change of the C ring, and the absence of an OH, which suggested that sulfate was located at C-3′ or C-4′ in **2**. The strong HMBC correlations of H_3_-7′/C-1′, C-5′, and C-6′ (δ_C_ 133.7), and H-5′/C-1′, C-3′, and C-4′ (Figure 3 and Supplementary Figure 13), and NOESY correlations between H_3_-7′ and H-6, H-5′ indicated that CH_3_-7′ was located at C-6′ (Figure 3 and Supplementary Figure 14). The substitution of hydroxyl at C-4′ and sulfate at C-3′ was deduced by the HMBC correlations of OH-4′ (δ_C_ 9.70) to C-3′(δ_C_ 127.5), C-4′, and C-5′ (Figure 3 and Supplementary Figure 13). Further detailed analysis of MS, HMBC, and NOESY spectrum (Figure 3 and Supplementary Figures 13–15) proved that **2** was a new compound and named it 3′-hydroxyalternariol 5-*O*-methyl ether-3′-*O*-sulfate.

The remaining 10 known compounds were identified as 3′-hydroxyalternariol 5-*O*-methyl ether (**3**), alternariol (**4**), alternariol-5-*O*-methyl ether (**5**), altenusiol (**6**), isoaltenuene (**7**), altenuene (**8**), altenusin (**9**), 5′-methoxy-6-methyl-biphenyl-3,4,3′-triol (**10**), dihydroalterperylenol (**11**), and alterperylenol (**12**), by comparison of their NMR, MS, and specific rotation data with those described in the previously published manuscript (Shigemori et al., 1998; Aly et al., 2008; Zhang et al., 2012; Kim et al., 2014; Hildebrand et al., 2015).

### Antibacterial Activity

The isolated compounds (**1–12**) were assessed *in vitro* for antibacterial activity against eight FBB, including six Gram-negative bacteria (*E. coli, P. mirabilis, V. parahaemolyticus, P. fluorescens, S. choleraesuis*, and *S. flexneri*) and two Gram-positive bacteria (*S. aureus* and *L. monocytogenes*). As shown in Table 2, Supplementary Figure 2, and Supplementary Figure 19, all compounds showed moderate antibacterial activity, with MIC values ranging from 15.63 to 125 μg/ml and MBC values ranging from 31.25 to 125 μg/ml. In terms of inhibiting Gram-positive bacteria, these compounds were far weaker than the tetracycline (positive control). However, they were close in terms of inhibiting and eliminating Gram-negative bacteria. Compounds 1 and 2 containing rare sulfate groups showed similar activity to analogues, indicating that sulfate groups or hydroxyl groups may not be necessary functional groups for antibacterial agents. This conclusion also provides a new idea for the subsequent research and development of this framework with low toxicity and high-efficiency antibiotics or preservatives, because it has been reported in the previous literature that quinolone can increase the toxicity of the compound (Shi et al., 2019).

**Table 2:** The antibacterial activity of compounds **1**–**12** (MIC and MBC in μg/ml, tetracycline used as a positive control, “NT” means No Test).

**Compound**	***S. aureus*** ***(ATCC25923)***		***L. monocytogenes*** ***(CICC21633)***		***E. coli*** ***(ATCC25922)***		***P. mirabilis*** ***(CMCC49005)***		***V. parahaemolyticus*** ***(ATCC17802)***		***P. fluorescens*** ***(ATCC13525)***		***S. choleraesuis*** ***(CICC13312)***		***S. flexneri*** ***(CMCC51571)***	
	**MIC**	**MBC**	**MIC**	**MBC**	**MIC**	**MBC**	**MIC**	**MBC**	**MIC**	**MBC**	**MIC**	**MBC**	**MIC**	**MBC**	**MIC**	**MBC**
1	62.5	250	> 250	> 250	62.5	250	31.25	125	62.5	125	31.25	62.5	62.5	125	15.63	> 250
2	62.5	125	> 250	> 250	62.5	125	31.25	125	62.5	125	31.25	62.5	62.5	125	15.63	> 250
3	31.25	125	NT	NT	31.25	125	NT	NT	NT	NT	NT	NT	31.25	125	NT	NT
4	62.5	125	125	> 250	62.5	> 250	31.25	125	62.5	125	62.5	> 250	62.5	> 250	> 250	> 250
5	62.5	250	> 250	> 250	62.5	> 250	31.25	125	62.5	125	62.5	> 250	> 250	> 250	> 250	> 250
6	31.25	125	31.25	62.5	31.25	125	31.25	125	31.25	125	31.25	31.25	31.25	62.5	31.25	> 250
7	> 250	> 250	> 250	> 250	31.25	125	31.25	125	62.5	125	62.5	> 250	31.25	125	31.25	> 250
8	62.5	125	NT	NT	62.5	125	NT	NT	NT	NT	NT	NT	> 250	> 250	NT	NT
9	62.5	125	> 250	> 250	62.5	250	31.25	125	62.5	125	62.5	62.5	62.5	125	15.63	> 250
10	62.5	125	NT	NT	31.25	250	> 250	> 250	> 250	> 250	NT	NT	31.25	125	NT	NT
11	62.5	125	31.25	62.5	31.25	250	31.25	125	62.5	125	31.25	62.5	62.5	125	31.25	125
12	62.5	125	31.25	62.5	31.25	125	31.25	125	62.5	125	31.25	62.5	31.25	125	31.25	125
Tetracycline	0.24	3.91	0.24	15.63	15.63	62.5	15.63	62.5	15.63	125	15.63	31.25	15.63	31.25	15.63	62.5

### Antibacterial Mechanism

#### Growth Curve Analysis

**Figure 4:**
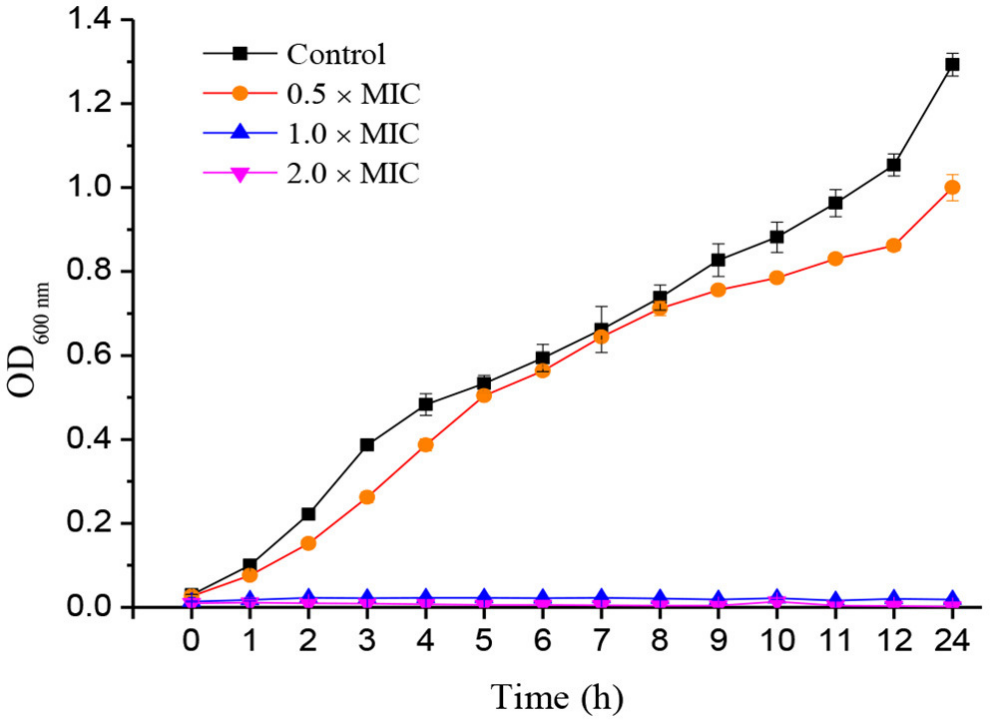
Growth curves of *Staphylococcus aureus* treated by compound **1**.

The growth curves of bacteria can reflect the effect of compounds on the growth of tested bacteria indirectly. According to the results of antibacterial assays, *S. aureus* was chosen to further investigate the growth curves of **1** at different concentrations (0.5 × MIC, 1.0 × MIC, and 2.0 × MIC). It can be seen that under the negative control and 0.5 × MIC treatment groups, the growth of *S. aureus* entered the logarithmic growth period after 1 h, and the number of bacterial colonies kept increasing within 24 h. When treated with 1.0 × MIC and 2.0 × MIC, the growth of *S. aureus* was almost completely stagnant, and there was no logarithmic period, which indicated that the bacteria were completely inhibited or even killed at the beginning of the drug treatment (Figure 4). The results showed that **1** exhibited very effective antibacterial activity against *S. aureus*, and its antibacterial effect was dose-dependent.

#### SEM Images of Bacteria Morphologies

**Figure 5:**
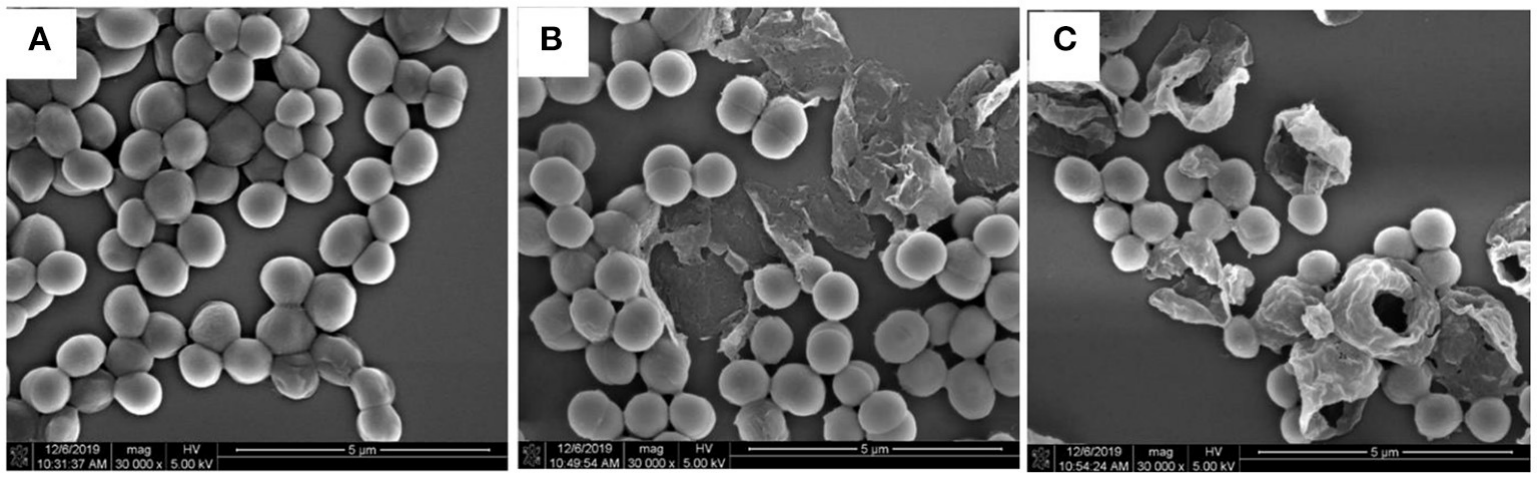
Scanning electron microscopy (SEM) images of *S. aureus* cells (× 30,000-fold). **(A)** Untreated *S. aureus*; **(B)** Treated *S. aureus* by compound **1** at 1.0 × MIC; **(C)** Treated *S. aureus* by compound **1** at 2.0 × MIC.

Scanning electron microscopy was used to observe the morphological change of *S. aureus* after treatment by **1**. In Figure 5A, the control bacteria cells preserved a typical staphylococcal arrangement structure, complete, smooth, and round cell surface, without obvious damage. In contrast, the cells of the 1.0 × MIC and 2.0 × MIC treatment groups were rough, wrinkled, irregular, and even with holes on the cell surface (Figures 5B,C). These observations indicated that the possible bacteriostatic mechanisms of **1** were to damage the cell membrane, thereby increasing its permeability.

#### FCM Analysis

**Figure 6:**
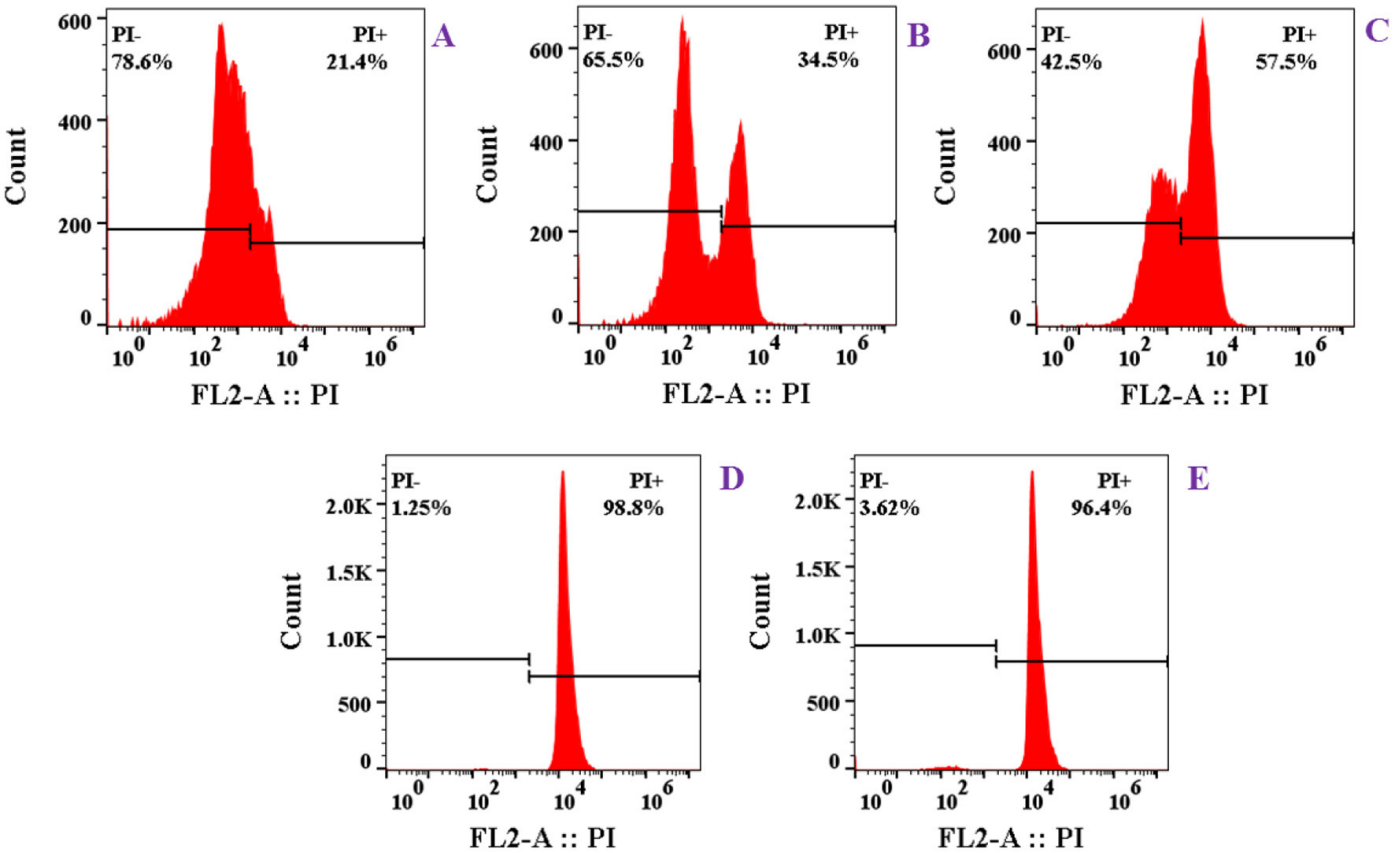
Effect of compound **1** on cell membrane permeability of *S. aureus* by flow cytometry (FCM) analysis: **(A)** control; **(B)** 0.5 × MIC; **(C)** 1.0 × MIC; **(D)** 2.0 × MIC; **(E)** 4.0 × MIC.

Propidium iodide is a kind of nucleic acid fluorescent dye, which cannot penetrate the intact cell membrane. However, if the cell membrane structure is destroyed and the membrane permeability increases, PI can bind to DNA nonspecifically. Therefore, the uptake of PI by cells can reflect the changes in cell membrane permeability indirectly. As shown in Figure 6A, the ratio of stained cells was only 21.4% without treatment. After incubation of *S. aureus* with **1** at different concentrations (0.5 × MIC, 1 × MIC, 2 × MIC, and 4 × MIC), the uptake amount of PI increased markedly and stained cells reached 34.5, 57.5, 98.8, and 96.4%, respectively (Figures 6B–E). These results showed that the cell membrane permeability of *S. aureus* was significantly changed by **1** in a dose-dependent manner.

## Conclusion

Infectious diseases caused by FBB are increasing every year, and it is very necessary to develop new antibacterial drugs. The secondary metabolites of marine fungi are a treasure trove of antibacterial drugs due to their novel structures, rich biological activity, and large output. In this study, three marine fungi, **F40**, **F46**, and **F49**, as the research object, were screened chemically and antibacterially. As a result, the *Alternaria* **F49** was selected for further separation because its PDB fermentation product exhibited chemical richness and strong antibacterial activity at a concentration of 500 μg/ml.

The genus *Alternaria* is widely distributed in plants, soil, and marine environments. It can produce abundant secondary metabolites, such as biphenyls (altenusin), dibenzopyones (alternariol, alternariol-9-methyl ether, isoaltenuene, altenuene), perylene quinones (dihydroalterperylenol, alterperylenol), and other conjugated compounds (alternariol-3-glucoside, alternariol-3-sulfate) (Puntscher et al., 2019). In addition to the well-known toxicity, the pharmacological activities of these compounds have also attracted the attention of pharmacists and chemists. Altenusin displayed remarkable neuroprotective effects and could prevent tau fibrillization *in vitro* (Chua et al., 2017; Hou et al., 2021). Alterperylenol exhibited strong antibacterial activity (MIC of 1.95 μg/ml) (Zhao et al., 2018). In summary, two new sulfate-modified dibenzopyrones (**1**, **2**) and 10 known compounds (**3**–**12**) were isolated from the screened marine fungus *Alternaria* sp. SCSIOS02F49. It is worth mentioning that almost all the compounds showed a broad spectrum against Gram negative and positive bacteria. At the same time, it was speculated that there is no obvious correlation between the substitution of sulfate and the antibacterial activity of compounds. Further, antibacterial mechanisms indicated that **1** could enhance the permeability of bacterial cell membranes. Our findings provided lead compounds for the development of low toxicity and high-efficiency antibiotics or microbial preservatives.

## Data Availability Statement

The original contributions presented in the study are included in the article/Supplementary Material, further inquiries can be directed to the corresponding authors.

## Author Contributions

YC conceived and designed the research, determined the structures, and wrote the manuscript. CL isolated the compound and tested the antibacterial activity. KK and LN provided the modification of this manuscript. YT conceived of and proposed the idea. All authors read and approved the manuscript and agreed to publish the manuscript.

## Funding

This work was supported by National Natural Science Foundation of China (42006094), the Education and Research Projects for Young and Middle-aged Teachers of Fujian Educational Department (JAT190237), and the School Fund of Fujian University of Traditional Chinese Medicine (No. X 2019007).

## Conflict of Interest

The authors declare that the research was conducted in the absence of any commercial or financial relationships that could be construed as a potential conflict of interest.

## Publisher's Note

All claims expressed in this article are solely those of the authors and do not necessarily represent those of their affiliated organizations, or those of the publisher, the editors and the reviewers. Any product that may be evaluated in this article, or claim that may be made by its manufacturer, is not guaranteed or endorsed by the publisher.
