# Supplementary material for: Two New Sulfate-Modified Dibenzopyrones With Anti-foodborne Bacteria Activity From Sponge-Derived Fungus Alternaria sp. SCSIOS02F49
Source: Front Microbiol. 2022 May 4;13:879674. doi: 10.3389/fmicb.2022.879674 (PMC9128073; doi:10.3389/fmicb.2022.879674)
Supplement: Supplementary file 1 [file Data_Sheet_1.docx]

Supplementary Material

**Two New Sulfate-Modified Dibenzopyrones with Anti-Foodborne Bacteria Activity from Sponge-Derived Fungus *Alternaria* sp. SCSIOS02F49**

**Yaping Chen^1^, Chuanna Liu^1^, Lihong Nan^1^*, Yongqi Tian^2^***

^1^College of Pharmacy, Fujian University of Traditional Chinese Medicine, Fuzhou, Fujian 350122, P. R. China

^2^ College of Biological Science and Engineering, Fuzhou University, Fuzhou, 350116, P. R. China.

*** Correspondence:** Lihong Nan (nlhong1152@163.com), Yongqi Tian ([tianyongqi@fzu.edu.cn](mailto:tianyongqi@fzu.edu.cn))

**Keywords: Sponge-derived fungi, *Alternaria* sp, sulfate-modified dibenzopyrones, anti-foodborne bacteria activity, antibacterial mechanism**

| **Contents** | **Pages** |
| --- | --- |
| **Figure S1**. The ^1^H NMR spectrum of **1** in DMSO | 3 |
| **Figure S2**. The ^13^C NMR spectrum of **1** in DMSO | 3 |
| **Figure S3**. The DEPT spectrum of **1** in DMSO | 4 |
| **Figure S4**. The HSQC spectrum of **1** in DMSO | 4 |
| **Figure S5**. The HMBC spectrum of **1** in DMSO | 5 |
| **Figure S6**. The NOESY spectrum of **1** in DMSO | 5 |
| **Figure S7**. The HRESIMS spectrum of **1** | 6 |
| **Figure S8**. The UV spectrum of **1** | 6 |
| **Figure S9**. The ^1^H NMR spectrum of **2** in DMSO | 7 |
| **Figure S10**. The ^13^C NMR spectrum of **2** in DMSO | 7 |
| **Figure S11**. The DEPT spectrum of **2** in DMSO | 8 |
| **Figure S12**. The HSQC spectrum of **2** in DMSO | 8 |
| **Figure S13**. The HMBC spectrum of **2** in DMSO | 9 |
| **Figure S14.** The NOESY spectrum of **2** in DMSO | 9 |
| **Figure S15**. The HRESIMS spectrum of **2** | 10 |
| **Figure S16**. The UV spectrum of **2** | 10 |
| **Figure S17**. The ^1^H NMR spectrum of **3** in DMSO | 11 |
| **Figure S18**. The ^13^C NMR spectrum of **3** in DMSO | 11 |
| **Figure S19**. The measurement of minimum bactericidal concentration (MBC) of compounds **1, 2** | 12 |
| **Table S1.** ^1^H and ^13^C NMR spectral data of compounds **2**, and **3** in DMSO-*d_6_*. | 13 |
| **Table S2.** The diameter of inhibition zone (DIZ) of compounds **1-12** | 14 |
| **Table S3.** The ITS region sequences of fungi **F40**, **F46**, and **F49** | 15 |


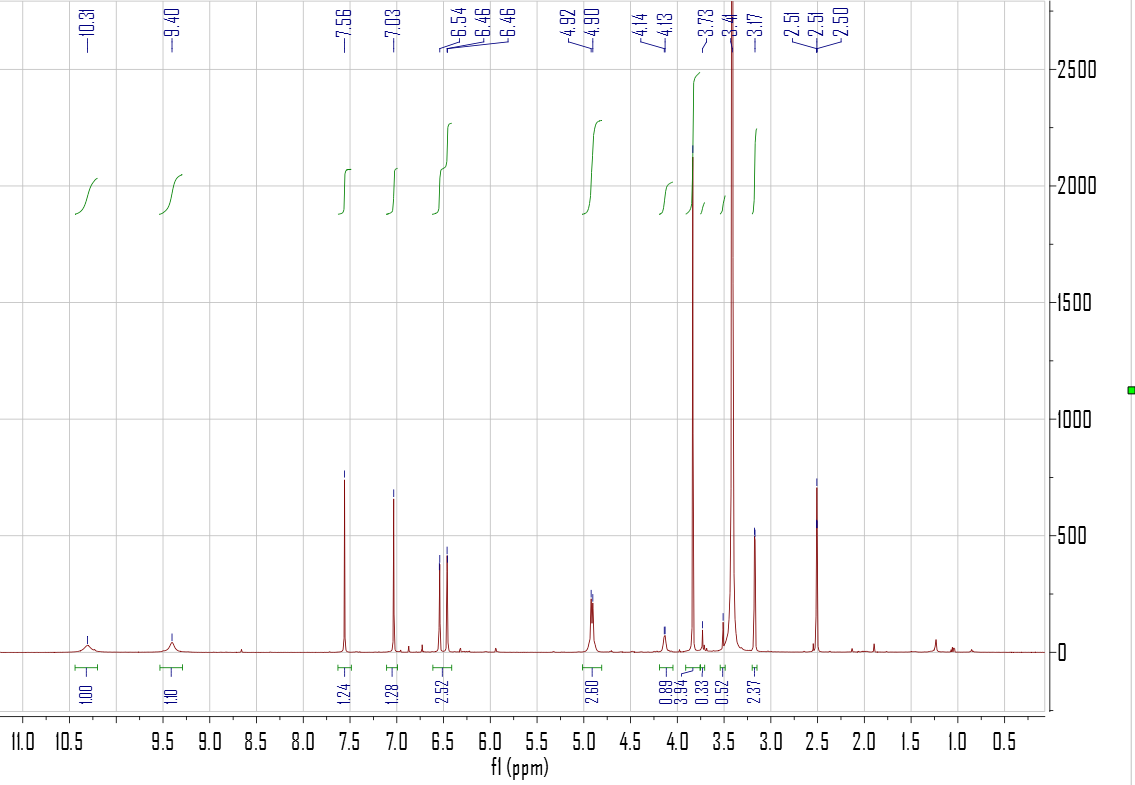


**Figure S1**. The ^1^H NMR spectrum of **1** in DMSO


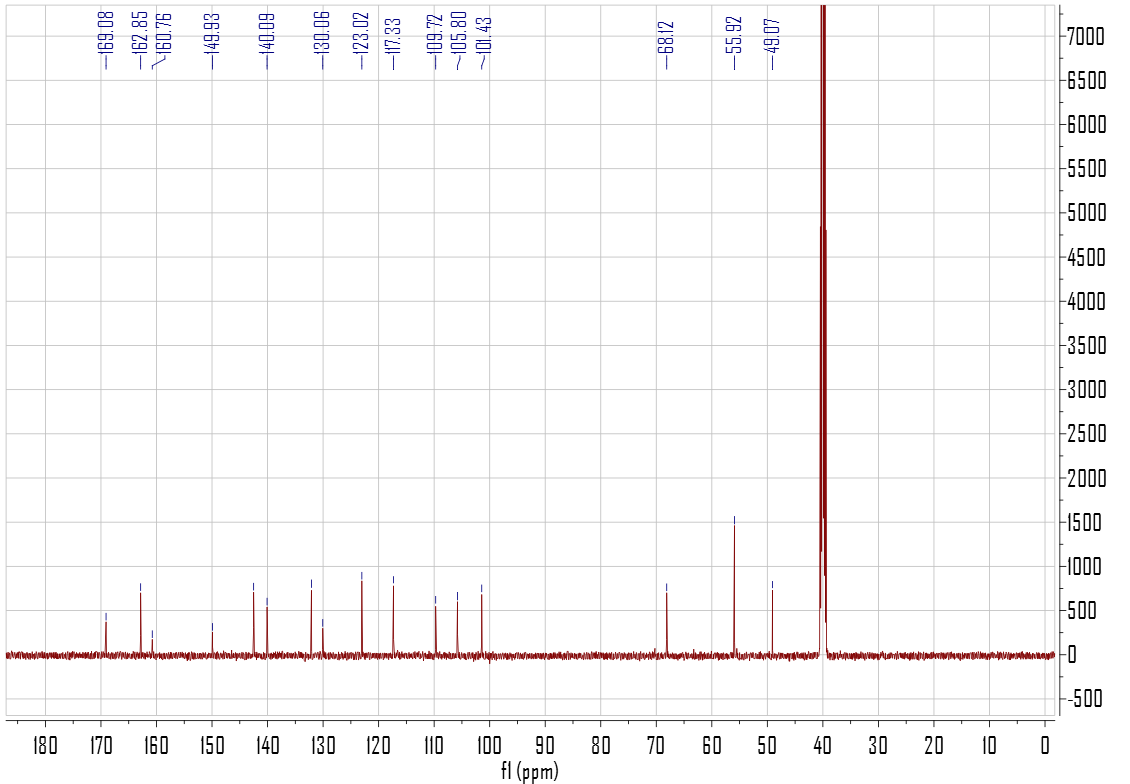


**Figure S2**. The ^13^C NMR spectrum of **1** in DMSO


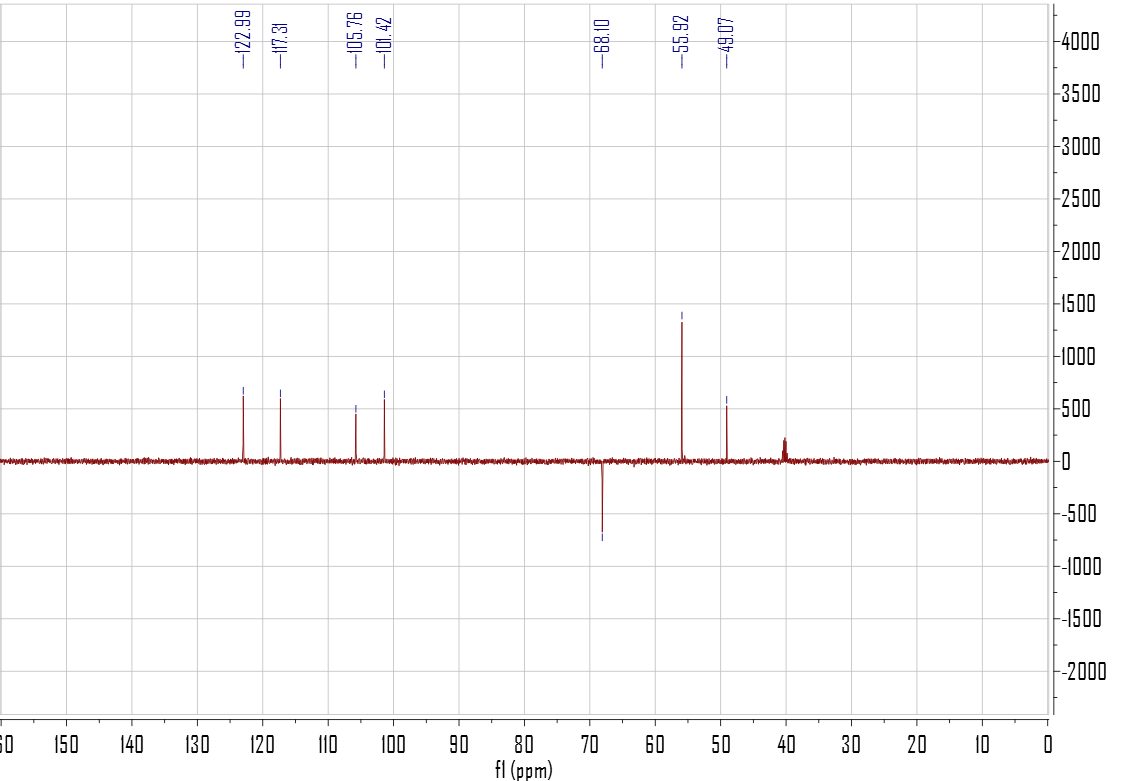


**Figure S3**. The DEPT-135 spectrum of **1** in DMSO


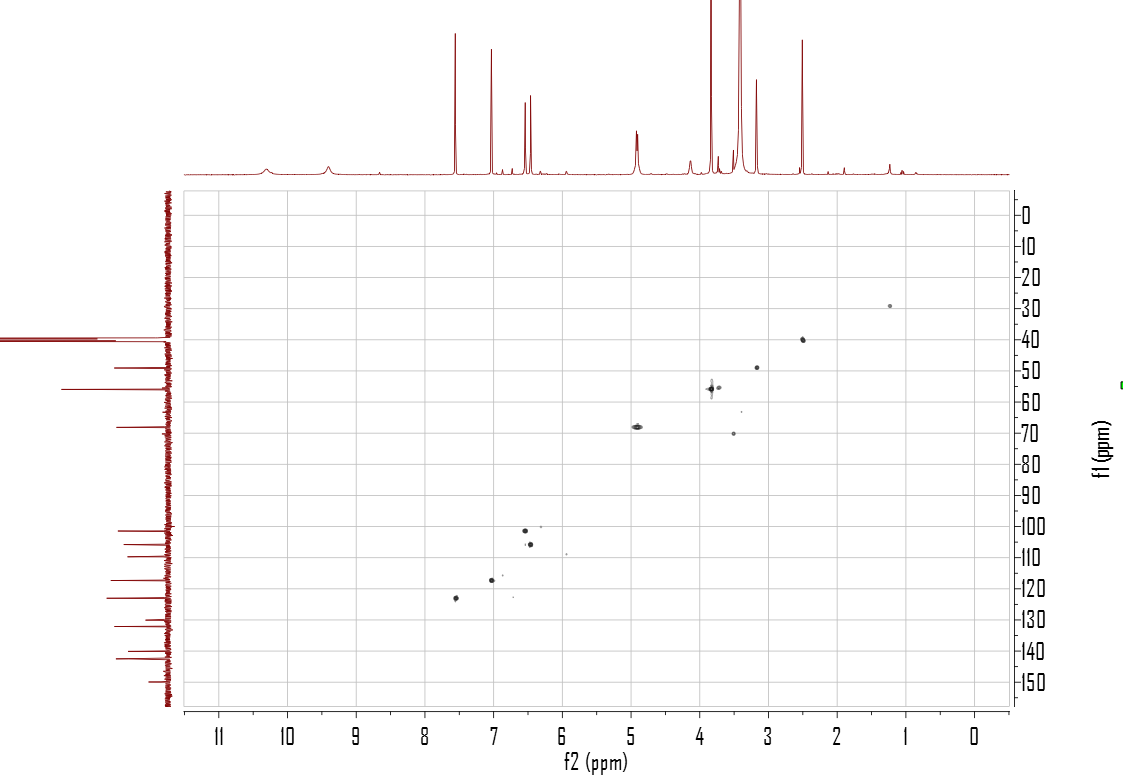


**Figure S4**. The HSQC spectrum of **1** in DMSO


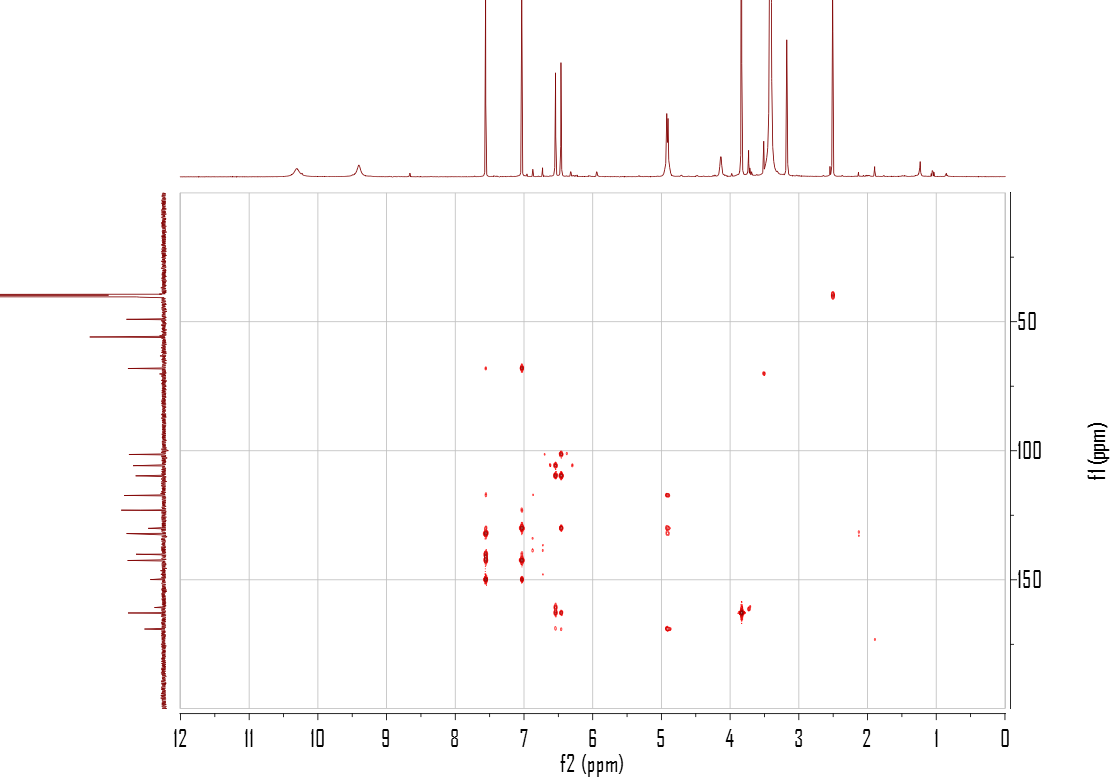


**Figure S5**. The HMBC spectrum of **1** in DMSO


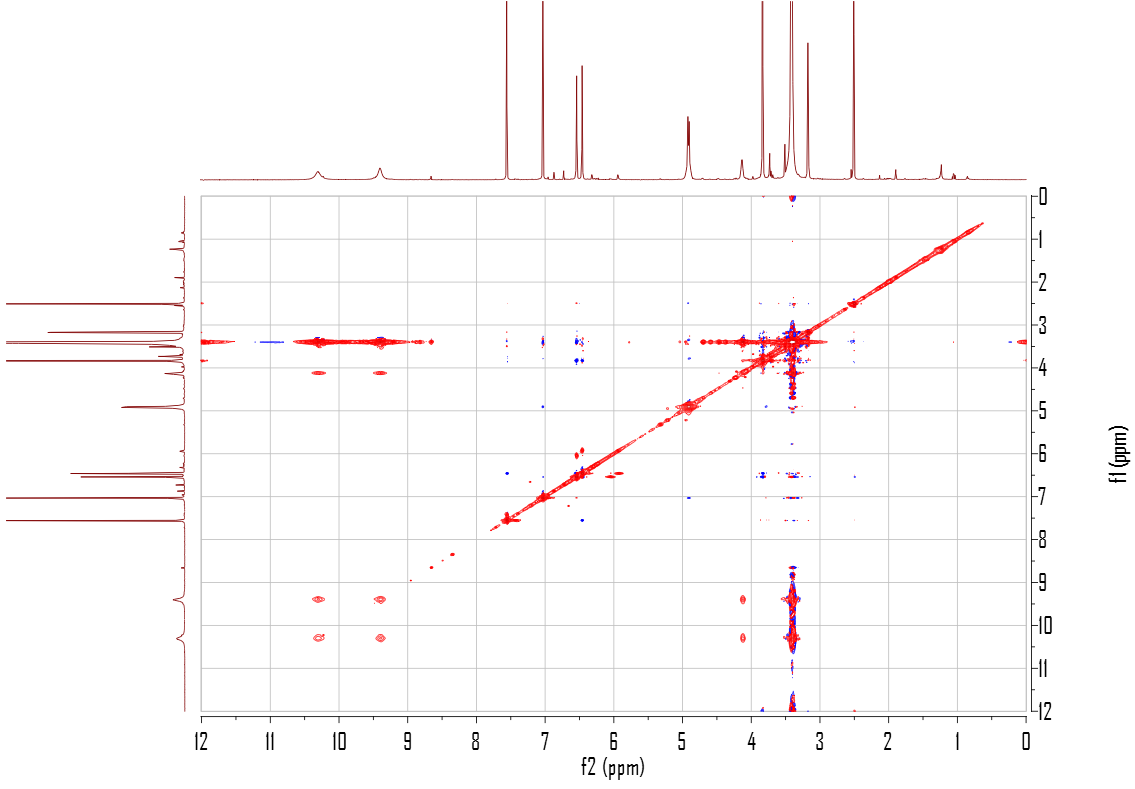


**Figure S6**. The NOESY spectrum of **1** in DMSO

**Figure S7**. The HRESIMS spectrum of **1**

**Figure S8**. The UV spectrum of **1** in methanol


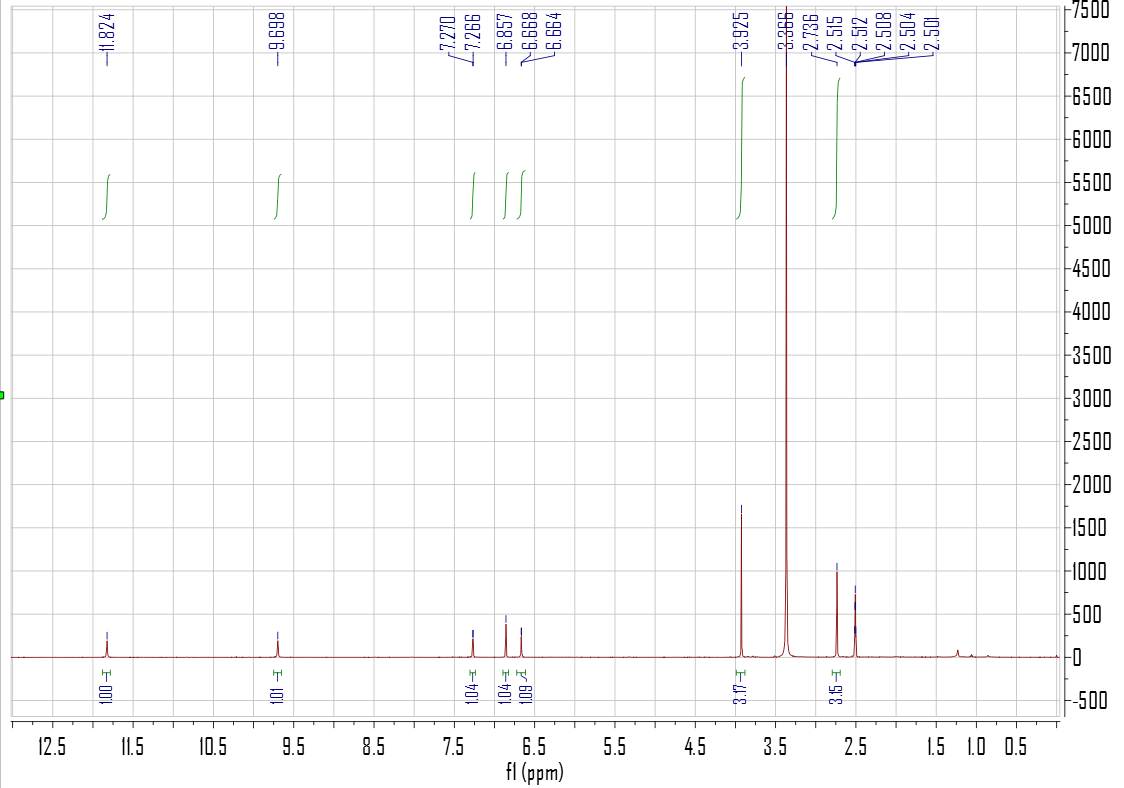


**Figure S9**. The ^1^H NMR spectrum of **2** in DMSO


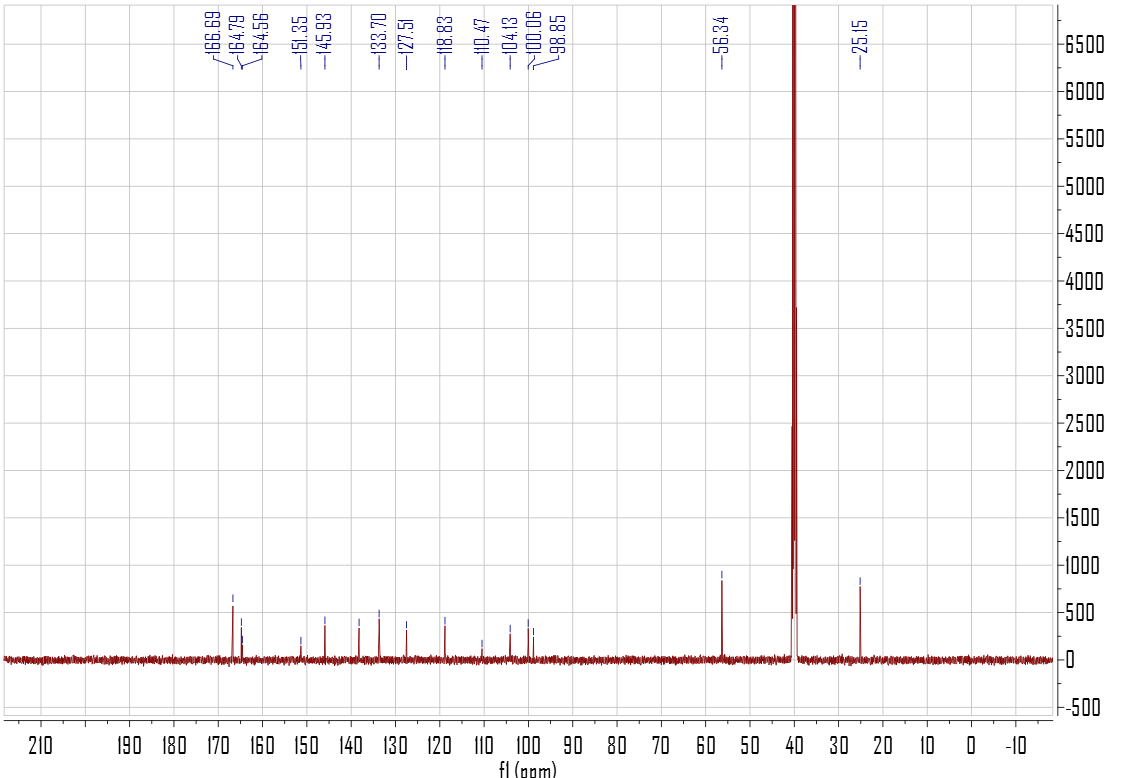


**Figure S10**. The ^13^C NMR spectrum of **2** in DMSO


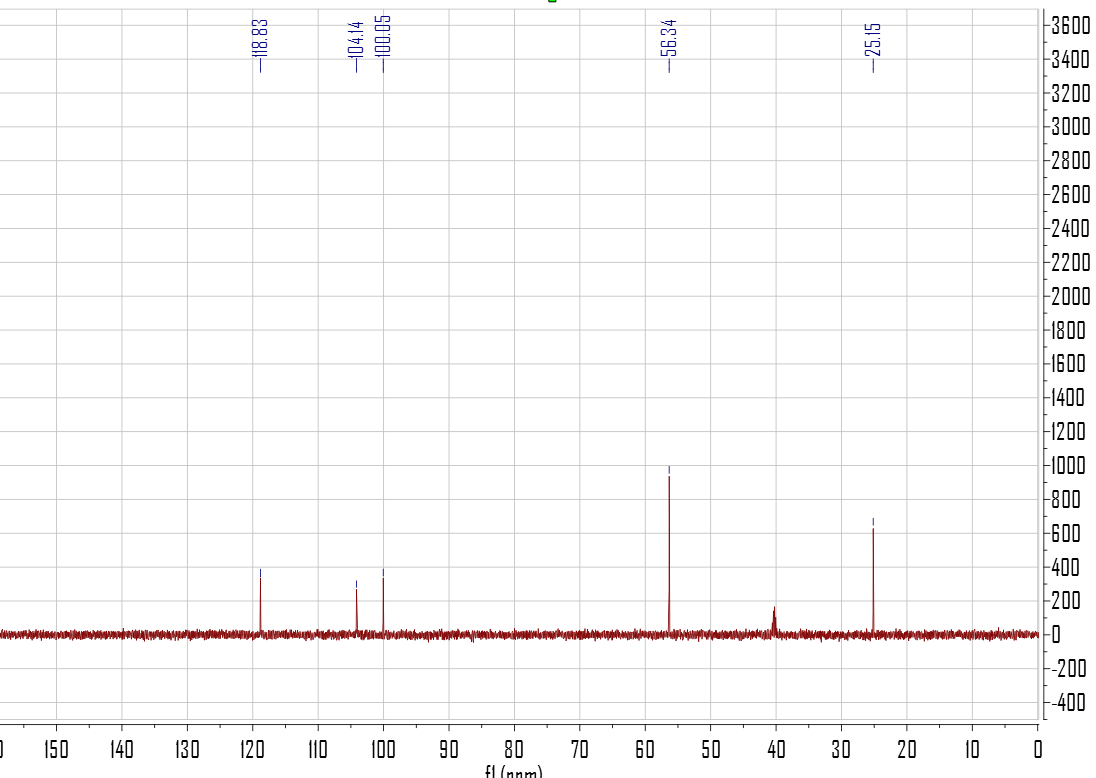


**Figure S11**. The DEPT-135 spectrum of **2** in DMSO


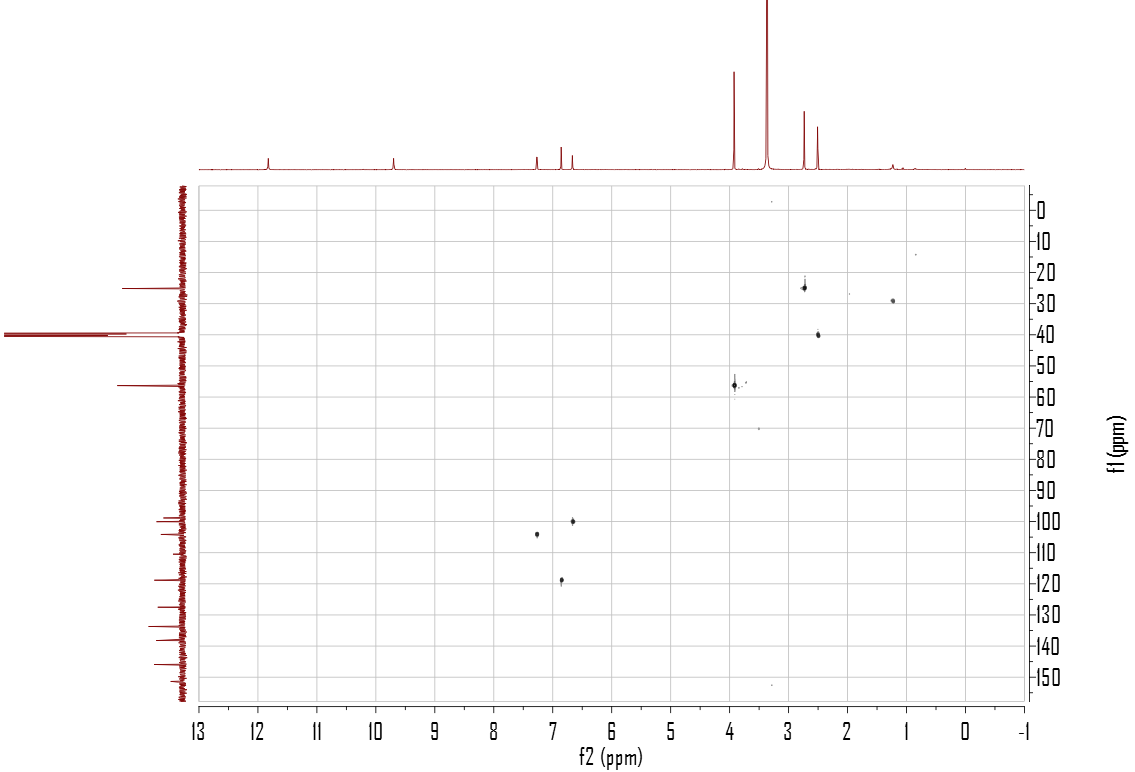


**Figure S12**. The HSQC spectrum of **2** in DMSO


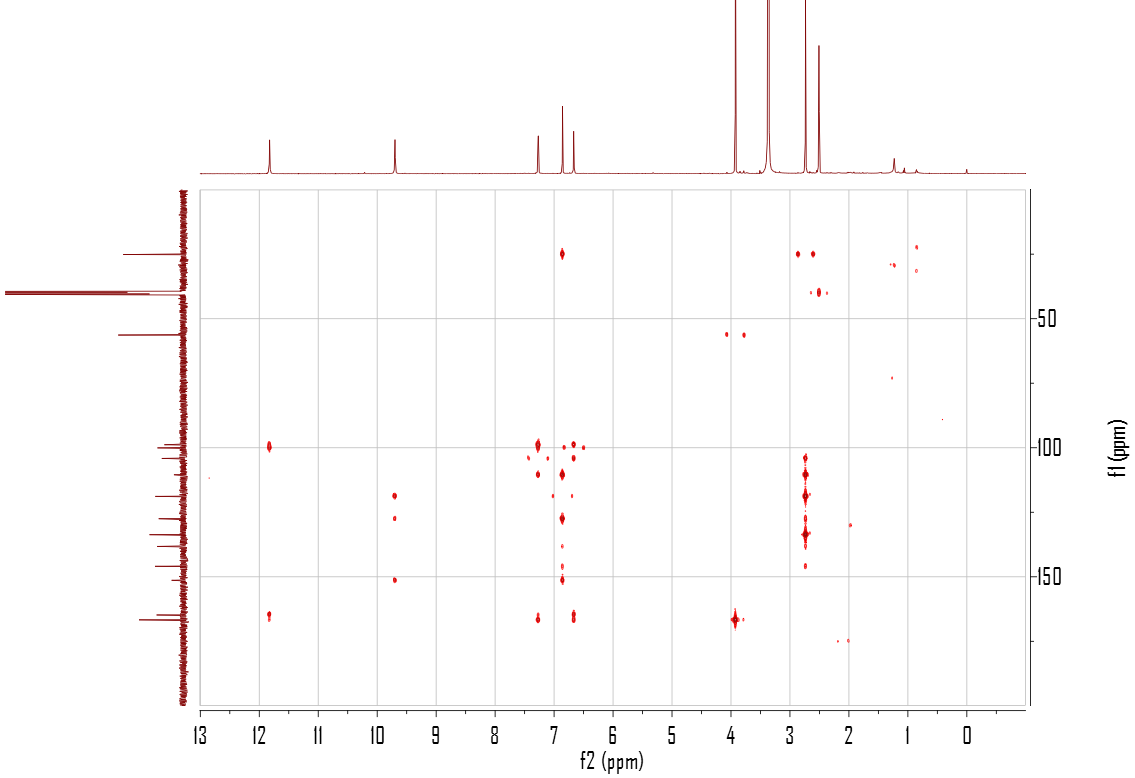


**Figure S13**. The HMBC spectrum of **2** in DMSO


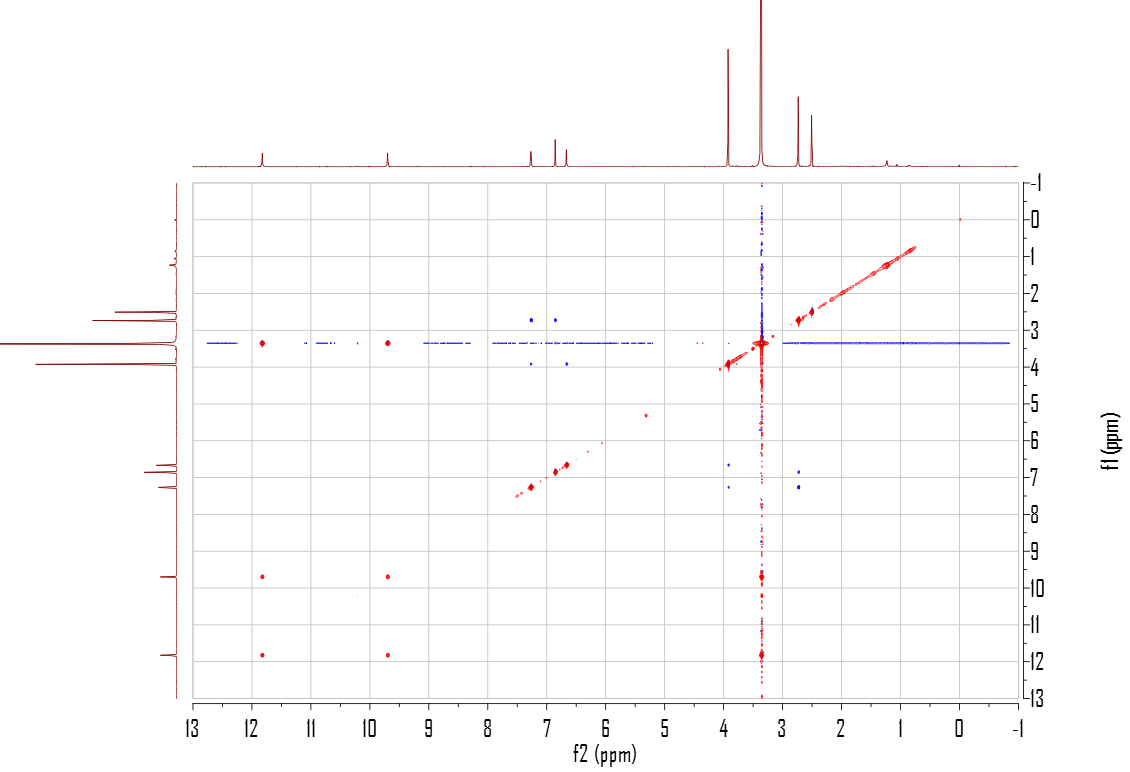


**Figure S14**. The NOESY spectrum of **2** in DMSO


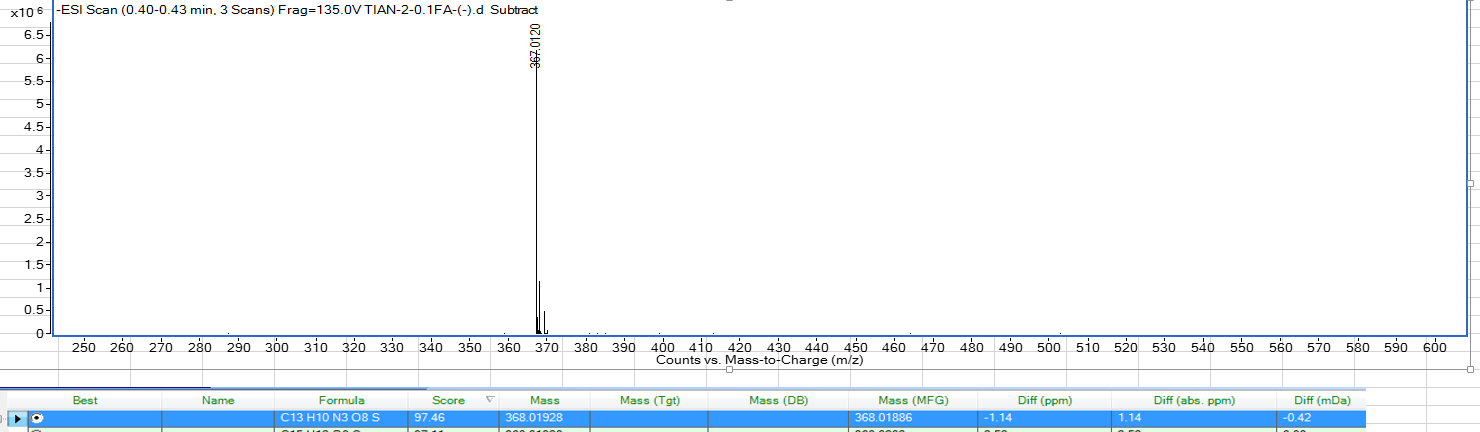


**Figure S15**.The HRESIMS spectrum of **2**

**Figure S16**. The UV spectrum of **2** in methanol


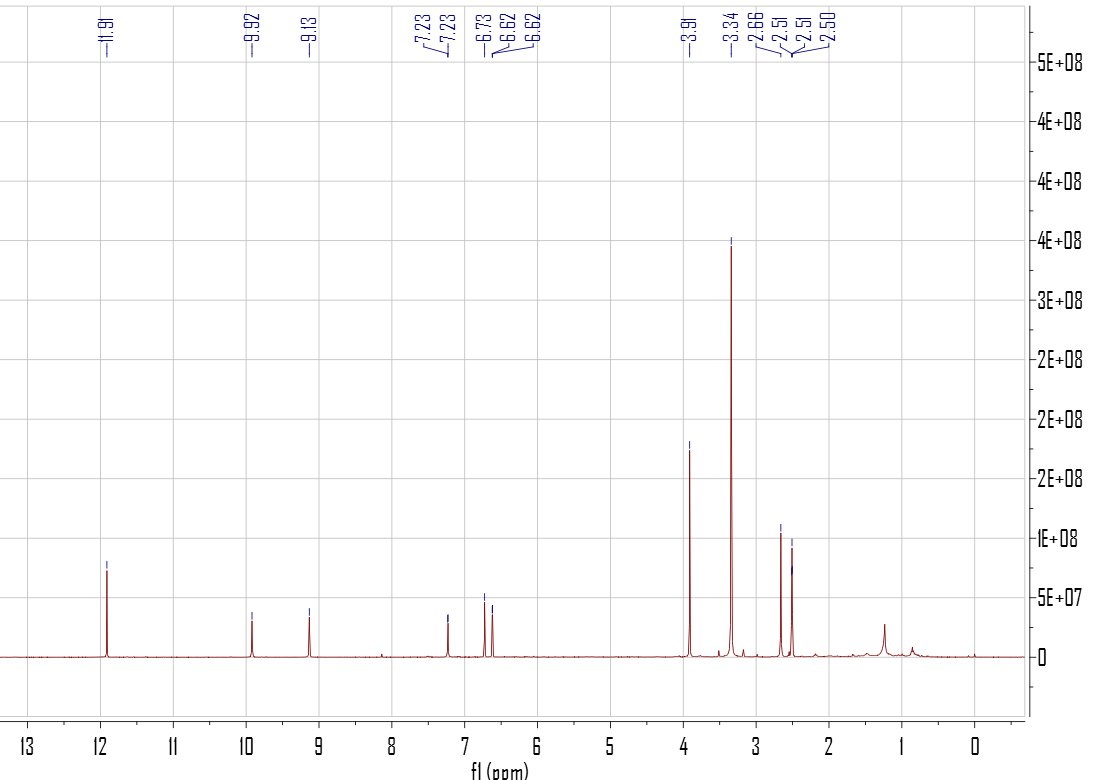


**Figure S17**. The ^1^H NMR spectrum of **3** in DMSO


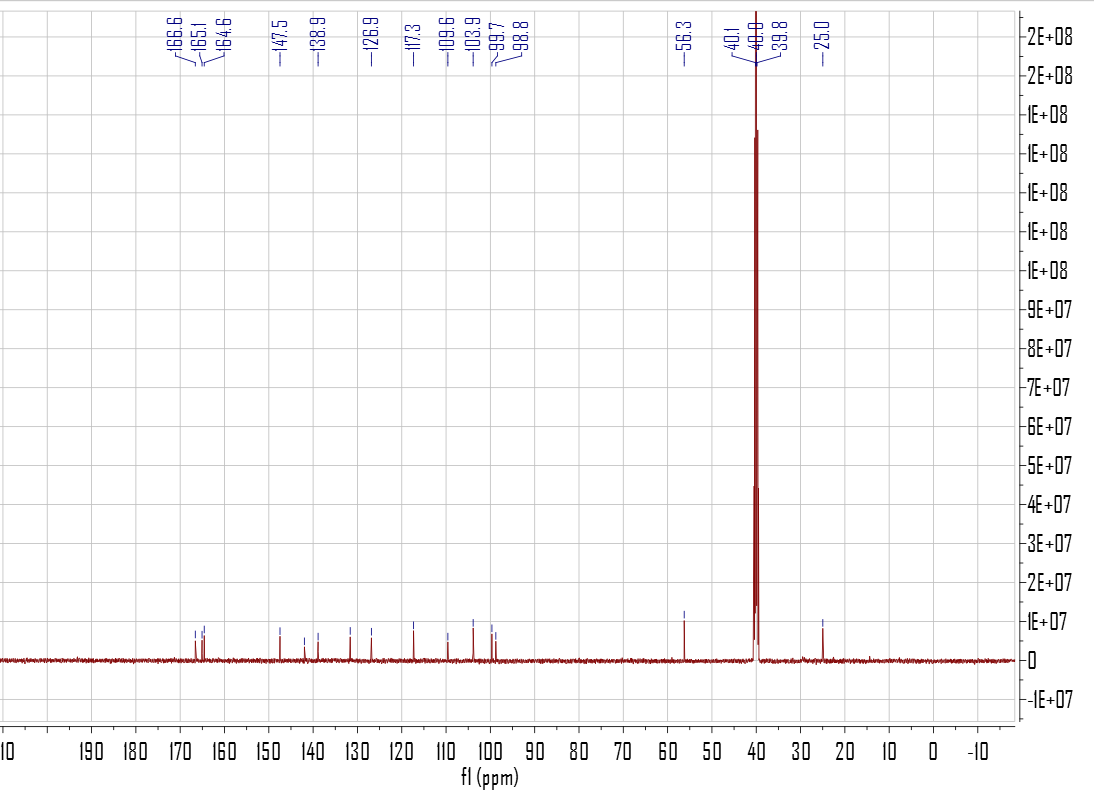


**Figure S18**. The ^13^C NMR spectrum of **3** in DMSO


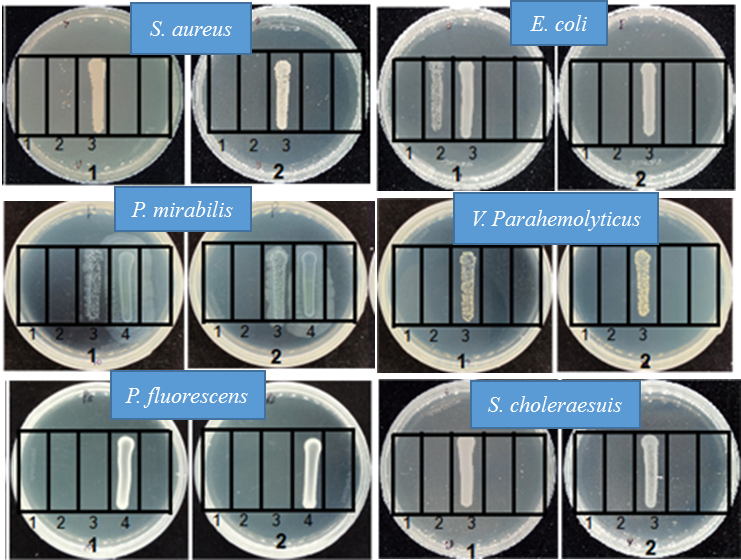


**Figure S19**. The measurement of minimum bactericidal concentration (MBC) of compounds **1, 2** against *S. aureus, E. coli*, *P. mirabilis, V. Parahemolyticus,* *P. fluorescens,* *S. choleraesuis* (The consecutive numbers “1~4” below the grid represented 250, 125, 62.5, 31.25 *μ*g/mL of the final concentration of compounds, respectively)*.*

**Table S1.** ^1^H and ^13^C NMR spectral data of compounds **2**, and **3** in DMSO-*d_6_*.

|  | **2** |  |  | **3** |  |
| --- | --- | --- | --- | --- | --- |
| Position | *δ*_H_, mult (*J* in Hz) | *δ*_c_, type | Position | *δ*_H_, mult (*J* in Hz) | *δ*_c_, type |
| 1 |  | 138.2, C | 1 |  | 138.9, C |
| 2 |  | 98.8, C | 2 |  | 98.8, C |
| 3 |  | 164.6, C | 3 |  | 164.6, C |
| 4 | 6.66, d (2.0) | 100.1, CH | 4 | 6.62, d (2.1) | 99.7, CH |
| 5 |  | 166.7, C | 5 |  | 166.6, C |
| 6 | 7.27, d (2.0) | 104.1, CH | 6 | 7.23, d (2.1) | 103.9, CH |
| 7 |  | 164.8, C | 7 |  | 165.1, C |
| 8 | 3.93, s | 56.3, CH_3_ | 8 | 3.91, s | 56.3, CH_3_ |
| 1' |  | 110.5, C | 1' |  | 109.6, C |
| 2' |  | 145.9, C | 2' |  | 142.3, C |
| 3' |  | 127.5, C | 3' |  | 126.9, C |
| 4' |  | 151.3, C | 4' |  | 147.5, C |
| 5' | 6.86, s | 118.8, CH | 5' | 6.73, s | 117.3, CH |
| 6' |  | 133.7, C | 6' |  | 132.4, C |
| 7' | 2.74, s | 25.1, CH_3_ | 7' | 2.66, s | 25.0, CH_3_ |
| 4'-OH | 9.70, s |  | 3'-OH | 9.13, s |  |
|  |  |  | 4'-OH | 9.92, s |  |
| 3-OH | 11.82, s |  | 3-OH | 11.91, s |  |

**Table S2.** The diameter of inhibition zone (DIZ) of compounds **1-12** (DIZ in mm; Methanol used as blank control; Tetracycline used as positive control.)

| **Inhibition zone (DIZ/mm)** | | | | | | | | |
| --- | --- | --- | --- | --- | --- | --- | --- | --- |
| **Com.** | ***S. aureus*** | ***S. choleraesuis*** | ***E. coli*** | ***L. monocytogenes*** | ***P. mirabilis*** | ***V. parahaemolyticus*** | ***P. fluorescens*** | ***S. flexneri*** |
| **1** | 6.67 (+) | 9.00 (+) | 7.00 (+) | 6.00 (-) | 7.00 (+) | 8.67 (+) | 7.67 (+) | 9.67 (+) |
| **2** | 7.00 (+) | 8.23 (+) | 7.00 (+) | 6.00 (-) | 10.00 (++) | 10.00 (++) | 11.00 (++) | 9.67 (+) |
| **3** | 10.00 (++) | 8.00 (+) | 7.00 (+) | / | / | / | / | / |
| **4** | 12.00 (++) | 7.00 (+) | 7.00 (+) | 8.33 (+) | 7.33 (+) | 14.67 (++) | 11.33 (++) | 6.00 (-) |
| **5** | 7.67 (+) | 6.67 (+) | 7.00 (+) | 6.00 (-) | 9.33 (+) | 8.00 (+) | 10.67 (++) | 6.00 (-) |
| **6** | 8.00 (+) | 9.00 (+) | 9.00 (+) | 9.33 (+) | 8.67 (+) | 13.00 (++) | 12.00 (++) | 10.00 (++) |
| **7** | 6.67 (+) | 8.00 (+) | 7.00 (+) | 6.00 (-) | 9.67 (+) | 9.00 (+) | 9.00 (+) | 7.67 (+) |
| **8** | 7.00 (+) | 10.33 (++) | 10.00 (++) | / | / | / | / | / |
| **9** | 8.00 (+) | 8.00 (+) | 7.33 (+) | 6.00 (-) | 12.00 (++) | 15.67 (+++) | 11.33 (++) | 8.67 (+) |
| **10** | 8.00 (+) | 8.00 (+) | 7.00 (+) | 6.00 (-) | 11.53 (++) | 14.30 (+++) | 10.20 (++) | 7.35 (+) |
| **11** | 6.33 (+) | 7.00 (+) | 7.00 (+) | 9.67 (+) | 8.33 (+) | 16.67(+++) | 8.33 (+) | 8.33 (+) |
| **12** | 11.67 (++) | 11.00 (++) | 9.00 (+) | 19.33 (+++) | 7.67 (+) | 21.33 (++++) | 15.67 (+++) | 8.00 (+) |
| MeOH | 6.00 (-) | 6.00 (-) | 6.00 (-) | 6.00 (-) | 6.00(-) | 6.00 (-) | 6.00 (-) | 6.00 (-) |
| TCY | 26.00 (++++) | 22.67 (++++) | 24.33 (++++) | 33.00 (++++) | 15.33 (+++) | 17.33 (+++) | 21.00 (++++) | 10.67 (++) |

Note: "-" no bacteriostatic activity (DIZ≤6 mm), "+" low bacteriostatic activity (6<DIZ<10 mm), "++" medium bacteriostatic activity (10≤DIZ<15 mm), "+++" strong bacteriostatic activity (15≤DIZ<20 mm), "++++" strong bacteriostatic activity (DIZ≥20 mm)

**Table S3.** The ITS region sequences of fungi F40, F46, and F49

| **Fungus code** | **Fungus storage number** | **ITS region sequences of fungus** | **genus** |
| --- | --- | --- | --- |
| F40 | MMPC NO. 5035 | CTGCTTCGGGCGCCACCTCCCACCCGTGACTACCTAACACTGTTGCTTCGGCGGGGAGCCCTCTCGGGGGCGCGCCGCCGGGGACTACTGAACTTCATGCCTGAGAGTGATGCAGTCTGAGTCTGAATATAAAATCAGTCAAAACTTTCAACAATGGATCTCTTGGTTCCGGCATCGATGAAGAACGCAGCGAACTGCGATAAGTAATGTGAATTGCAGAATTCAGTGAATCATCGAGTCTTTGAACGCACATTGCGCCCCCTGGCATTCCGGGGGGCATGCCTGTCCGAGCGTCATTGCTGCCCATCAAGCCCGGCTTGTGTGTTGGGTCGTCGTCCCCCCCGGGGGACGGGCCCGAAAGGCAGCGGCGGCACCGTGTCCGGTCCTCGAGCGTATGGGGCTTTGTCACCCGCTCGATTTAGGGCCGGCCGGGCGCCAGCCGACGTCCAACCATTTTTCTTCAGGTTGACCTCGGATCAGGTAGGGATACCCGCTGAACTTAAGCATATCAATAAGCGGAGGAA | *Aspergillus* |
| F46 | MMPC NO. 5036 | TTGCGTCGCGGCCCCCCTCCGGGGGCGGTTTGGGGTCCTCCCCTTCACGCGCGCACGACTGCATCCTTACTTTACGAGCACCTTCTGTTCTCCCTCGGCGGGGCAACCTGCCGTTGGAACCGAATAAACCCTTTTTTGCATCCAGCATTACCCGTTCCGAAACAAACAATCGTTACAACTTTCAACAATGGATCTCTTGGCTCTGGCATCGATGAAGAACGCAGCGAAATGCGATAAGTAGTGTGAATTGCAGAATTCAGTGAATCATCGAATCTTTGAACGCACATTGCGCCCCTTGGTATTCCGGGGGGCATGCCTGTTCGAGCGTCATCTACACCCTCAAGCTCTGCTTGGTGTTGGGCGTCTGTCCCGCCTCAGCGCGCGGACTCGCCCCAAATCCATTGGCAGCGGTCCTTGCCTCCTCTCGCGCAGCACATTGCGCTTCTCGAGGGGCTCCGGCCCGCGTCCAACAAGAAAACATTACCGTCTTTGACCTCGGATCAGGTAGGGATACCCGCTGAACTTAAGCATATCAAAAGCCGGAGGAA | *Didymellaceae* |
| F49 | MMPC NO. 5037 | ATCTCTCGGGGTTACAGCCTTGCTGAATTATTCACCCTTGTCTTTTGCGTACTTCTTGTTTCCTTGGTGGGTTCGCCCACCACTAGGACAAACATAAACCTTTTGTAATTGCAATCAGCGTCAGTAACAAATTAATAATTACAACTTTCAACAACGGATCTCTTGGTTCTGGCATCGATGAAGAACGCAGCGAAATGCGATAAGTAGTGTGAATTGCAGAATTCAGTGAATCATCGAATCTTTGAACGCACATTGCGCCCTTTGGTATTCCAAAGGGCATGCCTGTTCGAGCGTCATTTGTACCCTCAAGCTTTGCTTGGTGTTGGGCGTCTTGTCTCTAGCTTTGCTGGAGACTCGCCTTAAAGTAATTGGCAGCCGGCCTACTGGTTTCGGAGCGCAGCACAAGTCGCACTCTCTATCAGCAAAGGTCTAGCATCCATTAAGCCTTTTTTCAACTTTTGACCTCGGATCAGGTAGGGATACCCGCTGAACTTAAGCATATCAAAGCCCGGAGGAAA | *Alternaria* |
